# The predictors of health-enhancing physical activity among working women in Singapore two years into COVID-19: a cross-sectional study

**DOI:** 10.1038/s41598-022-26022-3

**Published:** 2022-12-13

**Authors:** Ellene Lim, Hadassah Joann Ramachandran, Joyce Biaw Theng Er, Pearlyn Ng, Wilson Wai San Tam, Ying Jiang

**Affiliations:** 1 Alice Lee Centre for Nursing Studies, Yong Loo Lin School of Medicine, National University of Singapore, Level 2, Clinical Research Centre, Block MD, 11,10 Medical Drive, Singapore, 117597 Singapore; 2 National University Heart Center, National University Health System, Singapore, Singapore; 3 Alexandra Hospital, National University Health System, Singapore, Singapore

**Keywords:** Population screening, Risk factors

## Abstract

Physical activity (PA) levels may have changed since the COVID-19 pandemic. However, these changes are not well understood. The study aimed to describe the PA level and examine the predictive factors of a health-enhancing PA level among working women in Singapore two years into the COVID-19 pandemic. We undertook a cross-sectional descriptive correlational study. Three hundred participants were recruited and completed the online questionnaire between October and November 2021. In the PA analysis of 217 participants, only 32.7% of the participants achieved a health-enhancing PA level, while 44.7% of the total sample sat for 7 h or more daily. In the univariate analysis, occupation, nationality, monthly income, and average daily sitting hours were significantly associated with a high PA level. The current mode of work, living arrangement, and health-promoting lifestyle profile II_physical activity score remained significant in both univariate and multivariate analyses. Participants who worked from home and stayed with their families were less likely to achieve a health-enhancing PA level than those who had a regular workplace and did not stay with their families. Working women with a health-promoting physically active lifestyle were likelier to achieve a health-enhancing PA level. The long daily sitting time and suboptimal health-enhancing PA participation underscore the need for health promotion initiatives for working women.

## Introduction

The World Health Organization (WHO) proclaimed a global pandemic in March 2020 due to the worldwide spread of the novel coronavirus (COVID-19)^1^. While people are reverting to their pre-pandemic lives, the pandemic has accelerated new lifestyle changes that are likely to endure beyond the pandemic and become the "new normal"^2^. These changes may have long-term effects on individuals' health behaviors.

Regular physical activity (PA) has been widely recognized to yield numerous positive health effects, including lowering the risk of chronic diseases^3^; alleviating symptoms of depression and anxiety; improving mood and emotional well-being^4^; and optimizing the functional integrity of the immune system while reducing the risk and severity of viral infections^5^. These findings have significant implications, especially in the COVID-19 pandemic^6^. Conversely, a sedentary lifestyle and physical inactivity are related to increased cardiovascular and metabolic risks and all-cause mortality^7^. In a recent move to increase the uptake of PA, the WHO now endorses a minimum of 150–300 min of moderate PA (previously 150 min), 75–150 min of vigorous PA (once 75 min), or some equivalent combination of both per week^8^.

Sociodemographic factors have been shown to impact PA levels. Occupations, mainly those physically demanding, significantly contribute to PA level^9^. Other factors, such as age, socioeconomic status, marital status, education level, ethnic group, and having a child, are frequently included in research examining the determinants of PA level^10^. While behavioral factors have also been shown to influence PA levels, an investigation of these differences has been limited in the literature. In a 2017 systematic review on the behavioral determinants of PA, commonly cited individual-level factors associated with PA were psychological variables (e.g., attitude, self-efficacy, stage of change), behavioral variables (e.g., screen time, previous activity history, and smoking), and socio-cultural variables (e.g., social support)^10^. More recently, Baughn et al.^11^ found that social isolation, engagement with employment or other work-related responsibilities, and fewer opportunities due to facility closures and public health restrictive guidelines were commonly reported factors by American adults aged 50 and older that either decreased or maintained their PA levels at six months into the COVID-19 pandemic.

Before the outbreak of COVID-19, Singapore's relatively high overall activity levels were primarily the result of commute-related activities due to its comprehensive and integrated public transport system and infrastructure^12^. However, this advantage has been diminished due to the abrupt changes in working and lifestyle behaviors brought about by government-implemented COVID-19 restrictions^13^. The introduction of remote and hybrid work arrangements, closure of public parks and outdoor sports facilities, and banning of dine-in meals saw many Singaporeans subscribe to online food deliveries and interact with friends and family via video calls^14^. The effect of this shift in activity levels and behavioral habits is expected to last into the ongoing epidemiologic transition in COVID-19^15^. The introduction of alternative working arrangements also disrupts the routine of many employed individuals. Working women, in particular, are more susceptible to these impacts. One study found that women engaged in significantly less PA compared to men due to COVID-19^16^. Besides work, women also tend to bear an enormous burden of addressing household needs and providing childcare^17^. The burden of household responsibility has intensified during the COVID-19 pandemic, especially with children undergoing home-based learning^14^. While working from home allows for greater scheduling flexibility, it blurs the boundaries between work, family, and personal life, making it difficult for working women to rest, let alone exercise regularly^17^. A local study in 2016 found that only 35.5% of working women in Singapore met the required weekly minutes of moderate-intensity PA for cardiovascular benefits^18^. Similarly, Win et al.^12^ reported that female Singaporeans aged 30 to 49, those with lower educational attainment, and those working full-time had the least amount of regular exercise. Other studies have observed a commensurate increase in obesity in this particular group, corroborating these findings^19^.

Presently, there is a dearth of evidence on the factors influencing alterations in PA levels and sedentary behaviors in Singapore. Given that social, economic, and cultural differences between countries affect how people adapt to and manage their circumstances during and after a pandemic, it is especially critical to understand these shifts in behaviors in the context of individual countries^20^. From a health promotion standpoint, gaining a better insight into the factors that help keep people active, mainly working women whose PA might be impacted by COVID-19-related lifestyle changes, can aid in conceiving targeted, evidence-based health-promoting interventions to improve women's health and mental well-being amidst and beyond the COVID-19 pandemic.

Thus, the aims of the study were: (1) to describe the PA levels among working women in Singapore; (2) to identify potential factors associated with the health-enhancing PA level, such as sociodemographic characteristics, exercise self-efficacy, health-promoting lifestyle, coping style, mental well-being, perceived social support, quality of life and perceived health; and (3) to determine the predictive value of these factors on health-enhancing PA level among working women in Singapore.

## Methods

### Study design and setting

This study adopted a cross-sectional descriptive correlational approach. Between October and November 2021, 300 participants were recruited from the National University Health System (NUHS), an integrated academic health system comprising a university and its affiliated hospitals. The study was reviewed and approved by the National University of Singapore Institutional Review Board (NUS-IRB-2021–634) and granted an exemption. All methods were carried out in accordance with relevant guidelines and regulations. The study was carried out using an anonymous online questionnaire; therefore, the documentation of informed consent was not required. Nevertheless, informed consent was obtained from all subjects. On the opening page of the online questionnaire, we have indicated the study's aims, and that participation is entirely voluntary. The participants had to consent by selecting the "I agree to participate" option to access the questionnaire and formally respond to it; otherwise, they did not have to answer any questions and would exit the page.

### Participants and data collection

The inclusion criteria were female participants: 1) aged 21–65; 2) residing in Singapore; 3) working either full-time or part-time; and 4) being able to read and understand English or Chinese. Exclusion criteria were those who were 1) pregnant; 2) suffering from a severe mental disorder that was not in remission (such as schizophrenia or bipolar disorder); 3) having a medical condition that prohibits regular physical activity (such as heart failure or congenital heart disease).

Eligible female staff working in NUHS were invited via email recruitment. A brief description of the study and a link to the questionnaire was sent to university faculty secretaries, who subsequently forwarded the email to their female departmental staff. The co-investigators of the study site forwarded the email invitation to eligible participants working in the two affiliated hospitals. Data were collected through a secure web-based platform (i.e., Qualtrics) that can be accessed via mobile phones or personal computers. Participants' responses to the questionnaires were recorded anonymously.

### Sample size calculation

The sample size was determined by the potential number of factors affecting PA levels in logistic regression. Based on the literature review, fifteen independent variables (i.e., exercise self-efficacy, mental well-being, health-promoting behaviors, coping style, social support, perceived health, education level, age, income level, occupations, marital status, number of children, screen time, presence of a medical condition, history of mental health condition) were anticipated as the potential predictors of PA^9,10,21,22^. An estimated 15 subjects were required for each independent variable^23^. Thus, a total of 225 participants were needed. To improve statistical power and to account for missing data and potential unknown predictors in the study, it was expected that a total of 300 participants would be required.

### Measures

#### Physical activity and sedentary behavior

PA and sedentary behavior were measured by the *International Physical Activity Questionnaire (IPAQ short form)*^24^. Participants reported the number of days and the minutes/hours of the day they did "vigorous," "moderate," and "walking" activities in the past seven days. The scale measured all physical activities associated with work, chores, commuting, and leisure. The data were treated and scored according to the IPAQ scoring protocol (November 2005 revision)^24^. Cases in which participants responded "don't know/not sure" were classified as missing data and excluded from the analysis. In the calculation of summary scores, only data with at least 10 min of activity were included, while responses of less than 10 min were recoded as “zero.” In addition, the walking, moderate, and vigorous variables exceeding 180 min per day were each truncated into 180 min per protocol^24^.

The amount of PA was calculated by weighting the energy requirements for each activity, defined in terms of the "metabolic equivalent of a task" (MET) (i.e., "vigorous PA" equals 8.0 METs, "moderate PA" equals 4.0 METs, and "walking" equals 3.3 METs), to give a MET-min/week score. Each participant's total MET-mins/week was computed by summing the MET-mins/week for all three types of PA. We categorized the total PA into “low,” “moderate,” and “high” categories based on the criteria of the IPAQ scoring protocol^24^ (Table 1).

**Table 1:** Three physical activity categories based on IPAQ.

Physical activity category	Cut-off levels
1. Low	-No activity is reported OR
	-Some activity is reported but not enough to meet Categories 2 or 3
2. Moderate	Either of the following 3 criteria
	-3 or more days of vigorous activity of at least 20 min per day OR
	-5 or more days of moderate-intensity activity and/or walking of at least 30 min per day OR
	-5 or more days of any combination of walking, moderate-intensity or vigorous-intensity activities achieving a minimum of at least 600 MET-minutes/week
3. High	Any one of the following 2 criteria
	-Vigorous-intensity activity on at least 3 days and accumulating at least 1500 MET-minutes/week OR
	-7 or more days of any combination of walking, moderate or vigorous intensity activities accumulating at least 3000 MET-minutes/week

Adapted from the guidelines for data processing and analysis of the international physical activity questionnaire (IPAQ)- short and long forms (November 2005).

The "high" category is recommended as a population target for health-enhancing PA, given that the IPAQ assesses total PA in all domains^24,25^. The "moderate" category in the IPAQ is a relatively low threshold if "total daily activity" is assessed. Most adults would achieve it through daily background activities, such as work, housekeeping, and family care^25^. Lee et al.^26^ found that while correlating with an objective measure, IPAQ-SF overestimated PA by an average of 84%. Despite this, the "high" category could be correctly identified and discriminated against from inactive participants, although the distinction between the other two categories was suboptimal^27^. Therefore, the current study used the “high” PA category as the cut-off for health-enhancing PA as per IPAQ scoring protocol^24^.

Additionally, the IPAQ contains a separate question to assess sedentary behavior, measured by time spent sitting on a typical day during the last seven days^24^.

#### Health-promoting lifestyle

A health-promoting lifestyle is any behavior an individual practices to control, maintain and improve their health. The 52-item *Health-Promoting Lifestyle Profile II (HPLP-II)* questionnaire was used to measure participants' health-promoting behaviors^28^. The HPLP-II consists of a full scale and six subscales to assess behaviors that promote a healthy lifestyle in the following dimensions: "spiritual growth," "interpersonal relations," "nutrition," "physical activity," and "stress management"^28^. Participants responded on a 4-point Likert scale from 1 (never) to 4 (routinely). The overall health-promoting lifestyle score was obtained by calculating the average of an individual's responses to all 52 items. Likewise, the subscale score was the average of the answers to the subscale items. A higher score indicated higher levels of health-promoting behaviors. The scale has an alpha coefficient of 0.943 for the total scale and 0.793 to 0.872 for the subscales^28^.

#### Self-efficacy for exercise

The *Self-Efficacy for Exercise (SEE)* scale was used to measure the participants' exercise self-efficacy^29^. The 9-item scale measures a person's confidence (from 0 to 10) in persevering to exercise despite obstacles. The reliability and validity of the scale have been established^29^.

#### Mental well-being

Participants' mental well-being was measured by the *Mental Health Continuum Short Form (MHC-SF)*^30^. The MHC-SF contains 14 items that measure an individual's level of emotional well-being (three items), psychological well-being (six items), and social well-being (five items). The scale has demonstrated good internal consistency and test–retest reliability^30,31^. Participants were asked how often they experienced each positive mental health symptom between "never" and "every day." Their responses were classified as "flourishing," "moderate," or "languishing" in terms of positive mental health^30^.

#### Perceived available social support

Perceived social support was assessed by the *Short Form of the Social Support Questionnaire (SSQ6)*^32^. The scale assesses two fundamental components of social support: (1) the number of persons available to turn to in times of need (the number score), and (2) the level of satisfaction with the available help in that specific situation (the satisfaction score)^32^.

#### Quality of life (QoL) and perceived health

The *EQ-5D* was used to assess QoL and perceived health^33^. The self-assessment scale measures an individual's health-related QoL in the following five dimensions: mobility, self-care, usual activities, pain/discomfort, and anxiety/depression. In addition, the scale has an EQ visual analog scale (VAS), which is used to record the individual's assessment of their overall health today^33^. The scale has been translated into other languages and validated in different countries, including Singapore^34^.

#### Coping styles

*The Brief-COPE Inventory*^35^ was used to measure participants' coping strategies to manage routine changes and stress. The Brief-COPE inventory is a 28-item self-report scale measuring 14 conceptually different coping reactions, namely "self-distraction," "active coping," "denial," "substance use," "use of emotional support," "use of instrumental support," "behavioral disengagement," "venting," "positive reframing," "planning," "humor," "acceptance," "religion" and self-blaming." The scale aims to examine coping in naturally occurring environments comprehensively. Participants answered each item as authentic as possible, from "1 = I haven't been doing this at all" to "4 = I've been doing this a lot"^35^. The brief COPE has been translated into several other languages and validated in different populations^36^.

#### Sociodemographic data

Participants' sociodemographic data such as age; marital status; occupation; the highest level of education; the number of children; occupations; type of housing and living arrangement; participants' weight and height (BMI); high-risk health behaviors such as smoking, and alcohol consumption; behavioral habits such as daily sleep hours and estimated daily screen time; and medical histories, such as current medical conditions and previous mental health history were collected as well.

### Data analysis

Statistical analysis was conducted using SPSS version 27 (SPSS Inc., Chicago, IL). Means, standard deviations, frequency, and percentage were used to describe the participants' sociodemographic characteristics and outcome variables. In logistic regression analysis, the dependent variable was the level of PA, categorized as "high" versus "others." Univariate logistic regression analysis was first conducted to delve into the possible sociodemographic and key correlated variables. Variables with a *p*-value < 0.1 in the univariate analysis were selected as independent variables (IVs) for the multivariate logistic regression analysis (Supplementary Table 1). The backward selection method based on Wald's statistics was used to select the final model. All statistically significant levels were set at *p* < 0.05.

## Results

### Sample characteristics of the participants

Table 2 illustrates the sociodemographic characteristics and behavioral variables of the participants. The participants were, on average, 33.73 years old, with almost three-quarters (67.3%) made of Chinese ethnicity, less than half (38.7%) married, and most (85%) had a university education or higher. Most resided in public housing estates with families (66.7%). Close to half (43.4%) of the participants reported having a flexible work arrangement, either working remotely from home or alternating between remote and in-office work. In the current sample, 144 participants (47.9%) were frontline healthcare workers, including physicians, nurses, allied health professionals, and patient care assistants. The sample's mean BMI was 22.85 kg/m^2^. On average, participants slept 6.47 h per day and spent an average of 8.64 h per day on screens. Of all the participants, 30 participants (10%) were former or current smokers, 65 participants (21.7%) had an alcohol consumption habit, and 72 participants (24.0%) reported having at least one medical problem.

**Table 2:** Characteristics of the participants (N = 300).

Characteristics	Total n (%)
**Age (years), mean (SD)**	33.73 (9.54)
**BMI, mean (SD)**	22.85 (4.58)
**Ethnicity**	
Chinese	202 (67.3)
Malay	17 (5.7)
Indian	18 (6.0)
Others (Caucasian, Filipino, Japanese, Javanese-Chinese, Myanmar, Vietnamese)	63 (21.0)
**Nationality**	
Singaporean citizens or PR	219 (73.0)
Non-singaporean, permit holder	81 (27.0)
**Marital status**	
Married	116 (38.7)
Single/ Widowed/Divorced/Separated	184 (61.3)
**Number of children**	
0	205 (68.3)
1 or 2	74 (24.7)
3 and above	21 (7.0)
**Highest education level**	
Secondary	6 (2.0)
ITE/Polytechnic (vocational training)	39 (13.0)
University and above	255 (85.0)
**Monthly income**	
≤ S$ 3499	101 (33.7)
S$ 3500 – S$ 4999	125 (41.7)
≥ S$ 5000	74 (24.7)
**Employment status,**	
Working on a full-time basis	293 (97.7)
Working on a part-time basis	7 (2.3)
**Years of working experience in Singapore, mean (SD)**	9.54 (0.56)
**Occupation**	
Physician	7 (2.3)
Nurse/assistant nurse/patient care assistant	133 (44.3)
Allied health care professional	4 (1.3)
Technician/lab technologist	10 (3.3)
Clerical staff / patient service associate	11 (3.7)
Administrator/manager/executive/ financial professionals	58 (19.3)
Teaching professionals	23 (7.7)
Others (procurement, researchers, research engineer, scientific officer, chemist)	54 (18.0)
**Current mode of work**	
Regular place of work	170 (56.7)
Remote/work from home	71 (23.7)
Hybrid work arrangement (alternate working remotely with being in the office)	59 (19.7)
**Type of housing**	
1-room / 2–3 room HDB^a^/ other rented place	94 (31.3)
4–5 room/Executive Maisonette HDB^a^	148 (49.3)
Private flat/Executive condominium/Landed property	58 (19.3)
**Living with**	
Alone	24 (8.0)
With family	200 (66.7)
With friends/others (landlord, housemates, tenants, colleagues)	76 (25.3)
**Medical conditions**	
1 medical condition	51 (17.0)
2 or more medication conditions	21 (7.0)
No medical condition	228 (76.0)
**History of mental health conditions**	
Anxiety	7 (2.3)
Depression	4 (1.3)
No history of mental health conditions	290 (96.7)
**Ex-smoker (Yes), n (%)**	23 (7.7)
**Current smoker (smoke in the past 4 weeks) (Yes)**	7 (2.3)
**Alcohol consumption (Yes)**	65 (21.7)
**Average hours of sleep per day in the past 4 weeks, mean (SD)**	6.47 (1.06)
**Average hours of screen time per day in the past 4 weeks, mean (SD)**	8.64 (4.07)

^a^Public housing developed by Housing & Development Board (HDB).

### Descriptive statistics of outcome variables

Table 3 summarizes descriptive statistics of the outcome variables. Of the 217 participants included in the PA analysis, less than a third of the participants (32.7%) achieved a high level of PA that produced health benefits. On the other hand, the participants sat for an average of 7.17 h per day. In addition, 36.3% reported feeling "slightly" to "severely" anxious or depressed. The mean perceived overall health score for the day reported by participants was 82.08 out of 100.

**Table 3:** Descriptive statistics for outcome variables.

Characteristics	Mean (SD)
**International physical activity questionnaire (short)**	
Total MET-minutes/week	2534.46 (2237.46)
median (IQR)	1930.00 (3235.50)
Levels of PA, n (%)	
Low	39 (13.0)
Moderate	107 (35.7)
High	71 (23.7)
“Don’t know/not sure.”	83 (27.7)
Average sitting hours per day	7.17 (3.49)
Sitting ≥ 7 h per day, n (%)	134 (44.7)
Sitting < 7 h per day, n (%)	109 (36.3)
“Don’t know/not sure,” n (%)	57 (19.0)
**Health-promoting lifestyle profile II**	
Total Scale	2.35 (0.39)
Health responsibility	2.04 (0.46)
Physical activity	2.15 (0.58)
Nutrition	2.35 (0.48)
Spiritual growth	2.57 (0.52)
Interpersonal relations	2.60 (0.48)
Stress management	2.36 (0.47)
**Self-efficacy for exercise scale**	4.35 (2.02)
**Mental health continuum short form**	
Total score	40.32 (14.27)
Categorical diagnosis, n (%)	
Languishing	29 (9.7)
Moderately mentally healthy	173 (57.7)
Flourishing	98 (32.7)
**Social support questionnaire (short form)**	
SSQN (number score)	2.71 (1.73)
SSQS (satisfaction score)	5.15 (0.84)
**EQ5D**	
Mobility (% of any problem), n (%)	26 (8.7)
Self-care (% of any problem), n (%)	0 (0.0)
Usual activities (% of any problem), n (%)	22 (7.3)
Pain/Discomfort (% of any problem), n (%)	95 (31.7)
Anxiety/Depression (% of any problem), n (%)	109 (36.3)
Health Score (VAS)	82.08 (14.42)
**Brief COPE inventory**	
Acceptance	5.71 (1.56)
Active coping	5.41 (1.52)
Behavioral disengagement	3.31 (1.37)
Denial	2.98 (1.26)
Emotional support	4.94 (1.67)
Humor	4.03 (1.72)
Instrumental support	4.93 (1.72)
Planning	5.25 (1.55)
Positive reframing	5.25 (1.58)
Religion	4.71 (2.16)
Self-blame	4.10 (1.72)
Self-distraction	5.44 (1.57)
Substance use	2.36 (0.97)
Venting	4.49 (1.44)

### Correlates of a high level of PA

The significant factors associated with a high level of PA in simple logistic regression are presented in Table 4. Ethnic Chinese (OR: 0.438, 95% CI: 0.241–0.797, *p* = 0.007) had lower odds of achieving high PA than non-ethnic Chinese participants. Singaporean and Singapore permanent residents (PR) (OR: 0.496, 95% CI: 0.265–0.930, *p* = 0.029*)* had lower odds of having high PA when compared to work permit holders. As opposed to those with a monthly income of more than S$ 5,000, those earning less than S$3,499 per month (OR: 2.176, 95% CI: 1.026–4.612, *p* = 0.043) had higher odds of high PA. Healthcare workers (OR: 2.705, 95% CI: 1.510–4.846, *p* < 0.001) had higher odds of having a high PA level than non-healthcare workers. Compared to those who needed to go back to the workplace, those who worked remotely or worked from home (OR: 0.192, 95% CI: 0.083–0.443, p < 0.001) and those who had hybrid work arrangement (OR: 0.382, 95% CI: 0.180–0.808, *p* = 0.012) had lower odds of achieving a high level of PA. In contrast to participants who were staying with friends or others, those who stayed with family (OR: 0.273, 95% CI: 0.141–0.528, *p* < 0.001) had lower odds of having a high level of PA. Participants with higher HPLPII_ physical activity scores (OR: 1.653, 95% CI: 1.035–2.643, *p* = 0.036) were more likely to have a high level of PA, while those who had longer sitting hours per day were less likely (OR: 0.898, 95% CI: 0.823–0.979, *p* = 0.014) in achieving a high level of PA.

**Table 4:** Significant factors associated with a high level of PA in the univariate analysis.

Variables	O.R. (95% CI)	*p-value*
**Ethnicity**		
Chinese	0.438 (0.241, 0.797)	0.007*
Non-chinese (Ref)		
**Nationality**		
Singaporean citizens or PR	0.496 (0.265, 0.930)	0.029*
Non-singaporean, permit holder (Ref)		
**Monthly income**		
≤ S$ 3499	2.176 (1.026, 4.612)	0.043*
S$ 3500 – S$ 4999	1.232 (0.596, 2.546)	0.573
≥ S$ 5000 (Ref)		
**Current mode of work**		
Remote/work from home	0.192 (0.083, 0.443)	< 0.001*
Hybrid work arrangement	0.382 (0.180, 0.808)	0.012*
Regular place of work (Ref)		
**Occupation**		
Healthcare workers (doctors, nurses/PCA, allied health)	2.705 (1.510, 4.846)	< 0.001*
Non-healthcare workers (Ref)		
**Living with**		
Alone	0.382 (0.126, 1.157)	0.089
With family	0.273 (0.141, 0.528)	< 0.001*
With friends or others (landlord, housemate, tenant, colleague) (Ref)		
**HPLPII_ physical activity**	1.653 (1.035, 2.643)	0.036*
**Average sitting hours per day**	0.898 (0.823, 0.979)	0.014*

### Predictors of a high level of PA

In the final data analysis phase, multivariate logistic regressions were performed for a high level of PA. The variables in the final model are shown in Table 5. Current work mode, living arrangement, and HPLPII_ physical activity emerged as significant predictors of high PA level. These factors explained 20.5% (Nagelkerke R^2^ = 0.205) of the variance.

**Table 5:** Predictors of determinants of a high level of PA in the final model.

Variables	O.R. (95% CI)	Wald	*p-value*
**Constant**	0.298	3.076	0.079
**Current mode of work**		10.189	0.006*
Remote/work from home	0.245 (0.098, 0.612)	9.080	0.003*
Hybrid work arrangement	0.435 (0.189, 1.00)	3.838	0.050
Regular place of work (Ref)	1.00		
**Living with**		5.788	0.055
Alone	0.483 (0.147, 1.582)	1.447	0.229
With family	0.389 (0.180, 0.842)	5.741	0.017*
With friends or others (landlord, housemate, tenant, colleague) (Ref)	1.00		
**HPLPII_ physical activity**	2.084 (1.201, 3.617)	6.812	0.009*
***Cox & Snell R***^***2***^*** : 0.146, Nagelkerke R***^***2***^*** : 0.205, Chi-square statistic: 31.920, p* < *0.001***			

## Discussion

This study explored PA levels among working women in Singapore and their influencing factors two years into the COVID-19 pandemic, a time that might have been sufficient to shape new behaviors and habits^37^. Worldwide, PA levels have generally declined since COVID-19 due to social restrictions and safe distancing measures^38^. Similarly, the proportion of residents in Singapore who manage to engage in satisfactory PA declined from 80.1% in 2019 to 76.4% in 2020^39^.

To boost health and functional capacity, the American College of Sports Medicine (ACSM) and the American Heart Association (AHA) recommends at least 150 min of moderate-intensity exercise or 60 min of vigorous exercise a week that are in addition to time spent performing daily living activities^41^. Given that the IPAQ assesses all domains of daily life, therefore, it was recommended to use a higher threshold as population targets for health-enhancing benefits^24^. The “moderate” category reflects the minimum PA guidelines (150 min of moderate PA or 60 min of vigorous PA a week), which is less than the ideal amount of PA. The most recent WHO guideline suggests that exceeding 300 min of moderate or 150 min of vigorous physical activity is associated with extra health benefits with no increased risk of harm^42^. New evidence also found that participation in 5000 MET minutes per week resulted in a significantly lower risk for cardiovascular disease modality compared to the recommended level of 750 MET minutes per week^43^.

We found that the proportion of working women engaged in health-enhancing PA was suboptimal, a finding close to another study before COVID-19 (32.7% vs. 35.5%)^18^, and lower than women in countries such as Canada (54.8%), China (56.2%), New Zealand (52.2%) and the USA (56.7%), while slightly higher than women in other Asian regions such as Hong Kong (28.6%), Japan (16.7%), and Taiwan (19.1%)^40^. On the contrary, the average daily sitting time exceeded 7 h, and 44.7% of our participants reported sitting for 7 h or more per day. Two meta-analyses indicated that a self-reported daily sitting time of 7 h significantly increased the risk of impaired health^44,45^. Our findings of sub-optimal health-enhancing PA engagement and prolonged daily sitting time among working women indicate a need to develop health promotion strategies tailored to this population in the post-COVID era.

Unexpectedly, our sample showed no statistically significant association between health-enhancing PA and self-efficacy for exercise, mental well-being, perceived social support, quality of life, or coping style (Supplementary file Table 1). Occupation, ethnicity, nationality, monthly income, and average sitting hours per day were significant factors associated with a high level of PA in univariate analysis. In contrast, the current mode of work, living arrangement, and HPLPII_physical activity remained significant in both univariate and multivariate analyses. They were predictors of health-enhancing PA in our sample.

Our study revealed that healthcare workers in the hospital were more likely to achieve a health-enhancing PA, which is in tandem with existing literature. Lindsey et al.^46^ found that essential workers maintained high PA levels primarily through their work compared to non-essential workers. Therefore, the higher odds of achieving a health-enhancing PA level among healthcare workers in this study may be attributable to the fact that they engaged in more work-related PA than non-healthcare workers.

Notably, we observed that less than half (47.6%) of the nursing staff achieved a health-enhancing PA level, despite their job requiring extended hours of standing or walking. This finding suggests that work-related PA alone may be insufficient to accumulate optimal PA levels for health-enhancing benefits, accentuating the importance of leisure time exercise in health outcomes^47^. Dorner et al.^9^ found that those in physically demanding occupations engaged in less PA during their leisure time and vice versa. Therefore, future studies can investigate how occupational and leisure time PAs influence health outcomes differently. We also need to examine different strategies to promote leisure time exercise among frontline nursing staff and employees in physically demanding occupations.

Non-Singaporean permit holders and non-ethnic Chinese participants were more likely to achieve a health-enhancing PA level. These findings contrast with studies conducted in Western populations, which identified that ethnic minorities – particularly African American women, American Indians, and Hispanic women – were prone to a lack of infrastructure to support their motivation to engage in PA compared to White women^48^. Further analysis revealed that the non-Singaporean and non-ethnic Chinese groups in our sample had a much larger proportion of healthcare workers (82.7% in non-Singaporean permit holders vs. 35.2% in Singaporean citizens or PR; 76.5% in non-ethnic Chinese vs. 34.2% in Chinese).

We found that participants with a monthly personal income ≤ SGD 3,499 (≈ 2,491 USD) were more likely to achieve a health-enhancing PA level than those with a monthly personal income ≥ SGD 5,000 (≈ 3,559 USD). This inverse relationship between income and PA levels appears specific to Singapore's population, as similar patterns were reported in other local studies^12,49^. In contrast, studies conducted elsewhere have identified a positive relationship between PA and income levels^50^. While this variation may be partly the result of the Singapore government's health promotion efforts to address the needs of lower-income families^51^, it merits further investigation. Lower-income individuals in Singapore may be more likely to engage in active transportation because public transportation is easily accessible. On the other hand, it is tempting to assume that individuals with higher incomes would have better access and resources to recreational facilities; understanding their decision-making processes in engaging in PA may offer some direction for future environmental and policy approaches in health-promotion efforts that target these groups of women.

In the current sample, those who reported longer daily sitting hours were less likely to achieve a health-enhancing PA level. Despite frequently being used interchangeably, sitting too much and less physical activity are distinct concepts^52^. One study argued that individuals could be sufficiently active based on PA recommendations while sitting excessively^53^. Other research has indicated that a decreased PA follows an increase in sitting or sedentary behavior^12,54^. Prolonged passive sitting has been linked to increased all-cause mortality, but PA can mitigate or negate such negative consequences^55^. However, a local study found that many employees remained unaware of sedentary behavior as an independent risk factor for chronic disease^56^.

After nearly two years of working from home due to the pandemic and having experienced the benefits of remote work, the current work mode will likely persist through the epidemic phase because of the technological investments and processes implemented in the workplace^57^. Working on-site or having a regular place of work predicted a health-enhancing PA level in our sample. Our result is not surprising because commuting, especially for work, is the most significant contributor to total PA for most Singaporeans^39^. However, increased screen time and reduced outdoor activity have been observed while working from home^54^. It is worth considering how this decrease in commute-related time can be filled by leisure-time PA rather than sedentary behavior. There remains an opportunity for the Singapore government to partner with corporate workplaces to provide protected and paid wellness breaks during work hours and offer discounts at off-site recreational facilities for those working remotely from home. Such investments in workplace wellness programmes to encourage PA may reap long-term returns in the form of a healthier workforce bearing reduced healthcare costs at the national level and improved employee productivity and morale at the corporate level^58^.

In the present study, living with friends or others predicted a health-enhancing PA level. Many working women reported inadequate support for household and childcare responsibilities and difficulties balancing family-related and societal expectations^48^. Therefore, those who live with friends may have fewer family obligations and more social support, which has been found to be strongly associated with changes in PA levels^59^.

Lastly, HPLPII_ physical activity was found to predict a health-enhancing PA level in our participants, i.e., for every 1-point increase in this subscale, participants were two times more likely to achieve a health-enhancing PA level. The HPLP-II emphasizes the self-initiated actions and personal views that maintain and enhance the individual's sense of well-being^28^. Our findings suggest that those who achieved a health-enhancing PA level may have prioritized their health-promoting behaviors, particularly physical activity, above other competing matters and engaged in these behaviors "often" or "routinely." The importance of self-prioritizing decisions and consistency appeared to outweigh other psychological factors, such as exercise self-efficacy or coping styles, which were not significantly associated with PA levels in the current sample. According to the self-determination theory, behavior is more likely to maintain if it reflects a person's values and is viewed as personally relevant^60^. This finding may shed some light on future interventions to promote PA among working women. In an Asian society, patriarchal values are deeply rooted in most aspects of the Singaporean population. Societal stereotypes in Singapore bind women to obligations at home^61^. Therefore, if PA does not imply personal relevance or is seen as less of a priority by working women, its maintenance would be harder to sustain.

COVID-19 has brought about new changes in lifestyle and PA habits. Health promotion strategies that promote women's active participation in PA may require strategies to boose their intrinsic motivation in prioritizing their physical health. On a larger scale, it is integral to simultaneously address the mental models arising from societal stereotypes to enhance the efficacy of supporting systems for women. Future interventions can explore novel ways to split PA and include them into the everyday routines of working women, especially for those that juggle between family and work responsibilities, so that the total PA required for health benefits can be accumulated. For example, by providing women-only fitness and exercise facilities adjacent to tutorials or enrichment classes, women can get some exercise while waiting for their children to attend class.

## Limitations

This study has several limitations. First, the cross-sectional nature of this study has resulted in our inability to establish causality in the relationships. Second, the Singapore population was not well-represented as participants were recruited from a university and its affiliated hospitals through convenience sampling, with an overrepresentation of nursing staff in the sample. In addition, our sample is much more educated than the general population. Third, self-reporting bias is inevitable as participants were required to assess their PA levels introspectively. Objective measures, such as a wearable device, will be the recommended method for determining the total PA level. In addition, this study is susceptible to recall bias as participants may erroneously respond to the self-administered questionnaires. Hence, the generalizability of findings from this current sample may be limited.

## Conclusion

Women make up a significant percentage of the workforce in Singapore. They face competing demands from work and a deep sense of family obligation, making them more vulnerable to suffering from poor physical and mental health. PA, such as walking, is known to have many health benefits and improve one’s overall well-being. Our study identified the factors and the predictors for health-enhancing PA in the context of two years into the COVID-19 pandemic. It offered recommendations to improve health-enhancing PA among working women and hoped that such findings could help formulate follow-up interventions to promote women's health and ultimately strengthen the resilience of the workforce in the post-COVID era.

## Supplementary Information

Supplementary Information.

## Data Availability

The data sets generated and/or analyzed in the current study are not publicly available, as this is a requirement consistent with the purpose and scope of the study at the time of the Institutional Review Board application, but are available from the corresponding author on reasonable request, a process that requires re-review and approval by the Institutional Review Board.
