# Supplementary material for: The predictors of health-enhancing physical activity among working women in Singapore two years into COVID-19: a cross-sectional study
Source: Sci Rep. 2022 Dec 13;12:21493. doi: 10.1038/s41598-022-26022-3 (PMC9747910; doi:10.1038/s41598-022-26022-3)
Supplement: Supplementary file 1 — Supplementary Information. [file 41598_2022_26022_MOESM1_ESM.docx]

**Supplementary Table 1. Screening variables for level of PA (high Vs other) in univariate logistic regression analysis**

| **Characteristics** | **O.R. (95% CI)** | ***P* value** |
| --- | --- | --- |
|  |  |  |
| **Ethnicity** |  |  |
| Chinese | 0.438 (0.241, 0.797) | 0.007** |
| Non-Chinese (reference) |  |  |
| **Nationality** |  |  |
| Singaporean Citizen or PR | 0.496 (0.265, 0.930) | 0.029** |
| Non-Singaporean, permit holder (reference) |  |  |
| **Marital status** |  |  |
| Married | 0.736 (0.409, 1.325) | 0.307 |
| Single/ Widowed/Divorced/Separated (reference) |  |  |
| **Number of Children** |  |  |
| 0 | 1.221 (0.358, 4.160) | 0.750 |
| 1 or 2 | 0.837 (0.226, 3.103) | 0.790 |
| 3 and above (reference) |  |  |
| **Highest education level** |  |  |
| Secondary School | 1.032 (0.092, 11.594) | 0.980 |
| ITE/Polytechnic | 1.032 (0.397, 2.684) | 0.949 |
| University and above (reference) |  |  |
| **Monthly income** |  |  |
| ≤ S$3499 | 2.176 (1.026, 4.612) | 0.043** |
| S$3500-S$4999 | 1.232 (0.596, 2.546) | 0.573 |
| ≥ S$5000 (reference) |  |  |
| **Employment status** |  |  |
| Working on full-time basis | 0.724 (0.118, 4.432) | 0.727 |
| Working on part-time basis (reference) |  |  |
| **Current mode of work** |  |  |
| Remote/work from home | 0.192 (0.083, 0.443) | <0.001** |
| Hybrid work arrangement | 0.382 (0.180, 0.808) | 0.012** |
| Regular place of work (reference) |  |  |
| **Occupations regrouped** |  |  |
| Healthcare workers (doctors, nurses/PCA, allied health) | 2.705 (1.510, 4.846) | <0.001** |
| Non-healthcare workers (reference) |  |  |
| **Type of Housing** |  |  |
| 1-room / 2-3 room HDB/other rented places | 2.326 (0.994, 5.443) | 0.052* |
| 4-5 room/Executive Maisonette HDB | 1.031 (0.461, 2.306) | 0.941 |
| Private flat/Executive condominium/Landed property (reference) |  |  |
| **Living with** |  |  |
| Alone | 0.382 (0.126, 1.157) | 0.089 |
| With family | 0.273 (0.141, 0.528) | <0.001** |
| With friends or others (landlord, housemates tenants, colleagues) (reference) |  |  |
| **Medical Conditions** |  |  |
| No | 0.893 (0.317, 2.514) | 0.830 |
| 1 medical condition | 1.364 (0.419, 4.435) | 0.606 |
| 2 and more medical conditions (reference) |  |  |
| **History of Mental Health Conditions** |  |  |
| No | 3.525 (0.425, 29.219) | 0.243 |
| Yes (reference) |  |  |
| **EQ5D** |  |  |
| Problem with mobility |  |  |
| No | 0.894 (0.340, 2.348) | 0.820 |
| Yes (reference) |  |  |
| Problem with usual activity |  |  |
| No | 0.457 (0.164, 1.271) | 0.134 |
| Yes (reference) |  |  |
| Problem with pain/discomfort |  |  |
| No | 0.743 (0.406, 1.360) | 0.336 |
| Yes (reference) |  |  |
| Problem with anxiety/depression |  |  |
| No | 1.189 (0.652, 2.169) | 0.572 |
| Yes (reference) |  |  |
| **Mental Health Wellbeing (Categorical Dx)** |  |  |
| Flourishing | 0.879 (0.335, 2.309) | 0.794 |
| Moderately Mentally Healthy | 0.651 (0.259, 1.639) | 0.363 |
| Languishing (reference) |  |  |
| **Ex-smoker** |  |  |
| Yes | 2.552 (0.825, 7.899) | 0.104 |
| No (reference) |  |  |
| **Current smoker** |  |  |
| Yes | 2.087 (0.288, 15.128) | 0.467 |
| No (reference) |  |  |
| **Alcohol** |  |  |
| Yes | 0.954 (0.478, 1.906) | 0.894 |
| No (reference) |  |  |
| **BMI** | 1.018 (0.962, 1.078) | 0.534 |
| **Age** | 1.0000 (0.970, 1.031) | 0.995 |
| **Sleep hours** | 0.955 (0.724, 1.259) | 0.742 |
| **Screen hours** | 0.965 (0.900, 1.035) | 0.324 |
| **Average sitting hours per day** | 0.898 (0.823, 0.979) | 0.014** |
| **Years of working** | 1.004 (0.975, 1.034) | 0.801 |
| **HPLPII Overall** | 1.620 (0.802, 3.274) | 0.179 |
| Health Responsibility | 1.641 (0.902, 2.986) | 0.105 |
| Physical Activity | 1.653 (1.035, 2.643) | 0.036** |
| Nutrition | 1.307 (0.727, 2.352) | 0.371 |
| Spiritual Growth | 1.322 (0.771, 2.267) | 0.310 |
| Interpersonal Relations | 0.993 (0.556, 1.776) | 0.982 |
| Stress Management | 1.211 (0.666, 2.203) | 0.530 |
| **MHC-SF Total** | 1.004 (0.984, 1.024) | 0.686 |
| Emotional Well-Being | 1.010 (0.926, 1.101) | 0.827 |
| Social Well-being | 1.007 (0.959, 1.057) | 0.784 |
| Psychological Well-being | 1.011 (0.969, 1.055) | 0.599 |
| **SSQN** | 1.012 (0.856, 1.197) | 0.887 |
| **SSQS** | 1.168 (0.838, 1.629) | 0.359 |
| **EQ5D Health Score** | 1.000 (0.980, 1.021) | 0.981 |
| **SEES** | 1.111 (0.967, 1.277) | 0.136 |
| **COPE** |  |  |
| Acceptance | 0.964 (0.801, 1.160) | 0.698 |
| Active coping | 1.000 (0.829, 1.206) | 0.998 |
| Behavioral disengagement | 1.088 (0.878, 1.348) | 0.441 |
| Denial | 0.975 (0.773, 1.229) | 0.831 |
| Emotional support | 0.983 (0.823, 1.174) | 0.847 |
| Humor | 1.025 (0.863, 1.217) | 0.781 |
| Instrumental support | 0.977 (0.824, 1.157) | 0.784 |
| Planning | 1.042 (0.859, 1.263) | 0.678 |
| Positive reframing | 1.064 (0.884, 1.281) | 0.510 |
| Religion | 1.034 (0.906, 1.179) | 0.624 |
| Self-blame | 1.027 (0.860, 1.226) | 0.768 |
| Self-distraction | 1.004 (0.840, 1.200) | 0.965 |
| Substance use | 0.744 (0.495, 1.119) | 0.156 |
| Venting | 1.099 (0.898, 1.344) | 0.360 |

**p* value <0.1, ** *p* value <0.05

The following variables with a *p* value of less than 0.1 in simple logistic regression analysis were included as the IVs in the multivariate logistic regression model: ethnicity, nationality, monthly income, current mode of work, occupation, type of housing, living with, average sitting hours per day, and HPLPII_physical activity subscale.
